# Landscape of somatic mutations in myeloproliferative neoplasm in Pakistani population

**DOI:** 10.12669/pjms.41.7.12129

**Published:** 2025-07

**Authors:** Mehreen Ali Khan, Suhaib Ahmed, Muhammad Arif Sadiq, Maryam Khan, Memoona Khan, Zaineb Akram

**Affiliations:** 1 Mehreen Ali Khan, MCPS, FCPS, Armed Forces Bone Marrow Transplant Centre, Rawalpindi, Pakistan; 2 Suhaib Ahmed, FCPS, PhD Department of Pathology, Riphah International University, Islamabad, Pakistan; 3 Muhammad Arif Sadiq, FCPS, PhD, Armed Forces Bone Marrow Transplant Centre, Rawalpindi, Pakistan; 4 Maryam Khan, MRCP, FCPS, Armed Forces Bone Marrow Transplant Centre, Rawalpindi, Pakistan; 5 Memoona Khan, FCPS, Armed Forces Bone Marrow Transplant Centre, Rawalpindi, Pakistan; 6 Zaineb Akram, PhD Department of Biological Sciences, National University of Medical Sciences, Islamabad, Pakistan

**Keywords:** CALR, JAK2 V617F, JAK2 exon 12, Myeloproliferative neoplasm

## Abstract

**Objectives:**

This study aimed to screen myeloproliferative neoplasm (MPN) patients for four known genetic variants to establish a diagnosis before treatment.

**Methods:**

This descriptive cross-sectional study was conducted at the Armed Forces Bone Marrow Transplant Center (AFBMTC), Rawalpindi, between January 2018 to January 2021. A total of 159 MPN patients were enrolled. Peripheral blood samples were screened for somatic variants in JAK2 V617F, JAK2 exon 12, CALR, and cMPL genes. The JAK2 V617F and cMPL mutations were analyzed using conventional PCR, while JAK2 Exon 12 and CALR mutations were analyzed using the fragment analysis technique. The gene scan data was interpreted by analyzing the electropherograms and the genotyping data sheet. The data were analyzed using the Statistical Package for Social Sciences (SPSS) version 25.0.

**Results:**

Out of a total of 159 MPN patients, 104 (65.4%) were males and 55 (34.6%) females. The median age of patients was 54 years (IQR: 38-64). 69 (43.4%) were diagnosed with primary myelofibrosis (PMF), 60 (37.7%) as polycythemia vera (PV), and 30 (18.9%) as essential thrombocytosis (ET). The frequency of the JAK2 V617F mutation in PV, ET, and PMF patients was 52.6%, 11.1%, and 37.3%, respectively. CALR mutation was observed only in 1 PMF and 5 (16.7%) ET patients. Additionally, cMPL mutation was not found among our patients.

**Conclusions:**

The four analyzed mutations are among the diagnostic criteria established by the World Health Organization, which enable a quick and reliable diagnosis of MPN.

## INTRODUCTION

In 1959, William Damaseek first used the term “myeloproliferative disease” in an editorial in the Blood journal.^1^ In 2008, the World Health Organization classified these diseases as Philadelphia-negative myeloproliferative neoplasms (Ph-ive MPNs), which included polycythemia vera (PV), essential thrombocytosis (ET), and primary myelofibrosis (PMF).^2^ They significantly impact quality of life and are associated with thrombosis and shortened survival. The majority of MPN cases harbor a mutation in codon 617 of Janus kinase 2 (JAK2), resulting in the replacement of valine with phenylalanine. These disorders are characterized by easy fatigability, pruritus, constitutional symptoms, abdominal microvascular symptoms, and an increased risk of thrombosis.^3^

Recently, genetic analysis has been increasingly used to improve disease diagnosis and optimize prognosis.^4^ Most of the data on managing these chronic MPNs is derived from the developed world, and diagnosing and managing these diseases in low- and middle-income countries poses a challenge. Although some studies have been conducted on the prevalence and clinicopathological features of MPNs in the Pakistani population^5^-^7^ there is limited information available on the frequency, demographics, treatment pattern, and outcome in Pakistani MPN patients. Moreover, PCR-based diagnostic techniques were previously used to detect genetic mutations in these neoplasms. We present a comprehensive clinical study on the molecular diagnosis of common mutations in MPNs using PCR and fragment analysis, which impacts the clinical course of disease and treatment outcomes.

## METHODS

This descriptive cross-sectional study was conducted at the Armed Forces Bone Marrow Transplant Centre (AFBMTC) from January 2018 to January 2021. It was carried out in accordance with the latest version of the Helsinki Declaration (adopted in 2013). A total of 159 patients were included in the study.

### Ethical Approval:

The study was approved by the Institutional Review Board of the institute (IRB-035/AFBMTC/Approval/2018, dated: February 25, 2025). Informed written consent was obtained from all the patients.

### Inclusion criteria:

- Diagnosed with PV, ET, or PMF based on their medical history, clinical examination, and the 2016 WHO diagnostic criteria,
- No treatment taken
- Consent form signed,

### Exclusion criteria:

- Secondary erythrocytosis, thrombocytosis or fibrosis
- Already on treatment
- BCR-ABL positive.^8^

Peripheral blood samples were collected for complete blood counts via an automated hematology analyzer, Sysmex KX21. Bone marrow aspirates (1 ml) and at least 2 cm trephine biopsy specimens were obtained from the posterior superior iliac spine of each patient, according to the guidelines of the International Council for Standardization in Hematology (ICSH)^9^. Each bone marrow aspirate sample was used for preparation of bone marrow slides followed by Giemsa staining and examination under microscope. \Core specimens were stained with hematoxylin (Bio Optica), eosin (SHURStain), and reticulin (in-house preparation with Merck reagents) stains. Additionally, 5 mL peripheral blood samples were taken in an EDTA tube for molecular analysis.

### Molecular Analyses:

All samples were screened for genetic mutations in JAK2 V617F, JAK 2 exon12, Calreticulin (CALR), and c-myeloproliferative leukemia (cMPL). JAK2 V617F and cMPL mutations were analyzed using conventional PCR products on polyacrylamide gel electrophoresis. JAK2 exon 12 and CALR mutations were analyzed using the fragment analysis technique on a genetic analyzer (ABI 3500) with POP-7 polymer and 50-cm capillary. Conventional PCR was performed by adding 100 ng DNA, 0.5 μM each primer (Table-I), and PCR master mix. Positive and negative controls were run with each sample. The fragment analysis results were analyzed using Gene Mapper five Software (Applied Biosystems). The data were interpreted by assessing the electropherograms and the genotype data sheet.

**Table-I:** Sequence of primers used.

JAK2V717F	Sequence 5’to 3’
Allele-specific forward primer	5’- AGCATTTGGTTTTAAAT TATGGAGTATATT -3’
Common reverse primer	5’- CTGAATAGTCCTACAGTGTTTTCAGTTTCA -3’
Forward control primer	5’- ATCTATAGTCATGCTGAAAGTAGGAGAAAAG -3’
JAK2 exon 12	Sequence 5’to 3’
Jak2 exon12-Forward primer	5’ FAM- CTCCTCTTTGGAGCAATTCA -3’
Jak2 exon12-Reverse primer	5’- TCCAATGTCACATGAATGTAAATC -3’
K539L allele-specific primer	5’ NED- GAACCAAATGGTGTTTCACTT -3’
CALR	Sequence 5’to 3’
CALRex9-F	5’ FAM- GAG GTG TGT GCT CTG CCT -3’
CALRex9-R	5’- GAGACATTATTTGGCGCGGC- 3’
cMPL W515L and W515K	Sequence 5’to 3’
MPL W515L forward primer	5’- GCCTGCTGCTGCTGAGGTT -3’
MPL W515K forward primer	5’- GCCTGCTGCTGCTGAGGAA3 -3’
MPL common reverse primer	5’- AAGTGGCGAAGCCGTAGGT -3’
Jak2 control-forward primer	5’- ATCTATAGTCATGCTGAAAGTAGGAGAAAG -3’
Jak2 control-reverse primer	5’- CTGAATAGTCCTACAGTGTTTTCAGTTTCA -3’

### Statistical Analyses:

The Statistical Package for Social Sciences software (IBM SPSS Statistics, New York, USA, version 25.0) was used to determine the frequency of qualitative variables and the mean, median, and standard deviation of quantitative variables. The *P*-value < 0.05 was considered statistically significant.

## RESULTS

**Fig.1:**
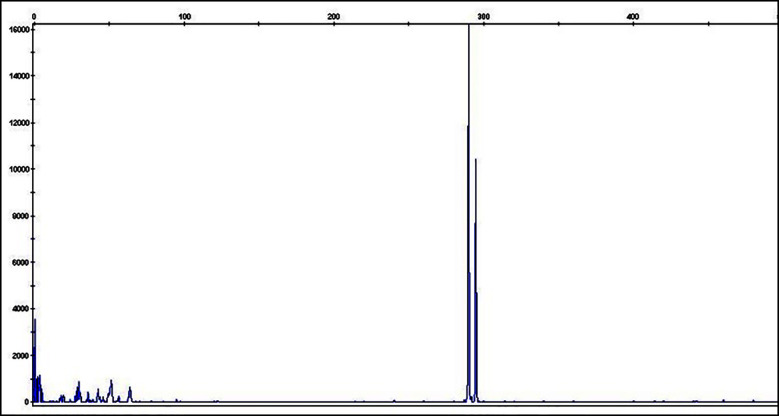
Pictogram of 5-bp insertion [ins]) identified during validation of the CALR fragment length analysis mutation assay.

A total of 159 MPN patients fulfilled the inclusion criteria, with 104 (65.4%) males and 55 (34.6%) females. The median age of patients was 54 years (IQR: 38-64). Among Ph-ive MPN patients, 69 (43.4%) were diagnosed with PMF, 60 (37.7%) with PV, and 30 (18.9%) with ET. All the patients were confirmed to be BCR-ABL negative before further study. The frequency of the JAK2 V617F mutation in PV, ET, and PMF patients was 51.7%, 13.3%, and 37.7%, respectively. CALR mutation was observed only in 1 (1.4%) PMF and 5 (16.7%) ET patients (Fig.1). Additionally, cMPL mutation was not found among our patients. JAK2 exon 12 was not detected in any of the patients. The frequency of mutations is given in Table-II.

**Table-II:** Frequency of mutations in MPN patients.

		JAK2 V617F		JAK2 exon 12		cMPL		CALR	
Disease	Total No. of Patients	Negative	Positive	Negative	Positive	Negative	Positive	Negative	Positive
		n (%)	n (%)	n (%)	n (%)	n (%)	n (%)	n (%)	n (%)
PV	60	29/60 (48.3)	31/60 (51.7)	60/60 (100)	0/60 (0)	60/60 (100)	0/60 (0)	60/60 (100)	0/60 (0)
ET	30	26/30 (86.7)	4/30 (13.3)	30/30 (100)	0/30 (0)	30/30 (100)	0/30 (0)	25/30 (83.3)	5/30 (16.7)
PMF	69	43/69 (62.3)	26/69 (37.7)	69/69 (100)	0/69 (0)	69/69 (100)	0/69 (0)	68/69 (98.6)	1/63 (1.4)
Total	159	98	61	159	0	159	0	153	6
P-value		0.001		0.15		---			0.0001

***Note:*** Chi-square test was applied. ***P* < 0.05** was statistically significant.

In Table-III, compares different demographics among PV, ET, and PMF patients. Common symptoms among MPN patients included generalized weakness (38.4%), abdominal discomfort (27.7%), pallor (22.6%), headache (11.3%), dizziness (9.4%), fever (8.2%), and clinically confirmed infections (8.8%), while bleeding and bruising were also observed in 7.5% and 5% of MPN patients, respectively. The mean blood counts at presentation in PV, ET, and PMF for both genders and different age groups are given in Tables IV and V, respectively. Splenomegaly and hepatomegaly were recorded in 49.7% and 25.8% of the patients, respectively, with median spleen and liver sizes of 6 cm (IQR: 3–12) and 3 cm (IQR: 2-5). respectively. Other clinically relevant comorbidities observed were ischemic heart disease (IHD) in 8.2% of patients and cerebrovascular accident (CVA) in 7.5%.

**Table-III:** Association of demographics and disease characteristics of MPN patients.

Patient demographics and disease characteristics		Disease			
		PV	ET	PMF	
		n (%)	n (%)	n (%)	P-value
Age Groups	1–15 Years	0 (0)	0 (0)	4 (5.8)	0.003
	> 15–30 Years	2 (3.3)	8 (26.7)	5 (7.2)	
	> 30–50 Years	24 (40)	7 (23.3)	19 (27.5)	
	> 50 Years	34 (56.7)	15 (50)	41 (59.4)	
Gender	Females	18 (30)	12 (40)	25 (36.2)	0.59
	Males	42 (70)	18 (60)	44 (63.8)	
Marital Status	Bachelor	1 (1.7)	5 (16.7)	9 (13)	0.02
	Married	59 (98.3)	25 (83.3)	60 (87)	
Phlebotomy	Nil	1 (1.7)	27 (90)	63 (91.3)	0.0001
	Upto 5	41 (68.3)	3 (10)	4 (5.8)	
	More than 5	18 (30)	0	2 (2.9)	
Pregnancy	Yes	0 (0)	1 (3.3)	0 (0)	0.17
	Did not conceive since diagnosis	7 (11.7)	3 (10)	5 (7.2)	
	Not Applicable	53 (88.3)	26 (86.7)	64 (92.7)	
BMA Trephine	Reticulin unremarkable	16 (26.7)	14 (46.7)	0 (0)	0.0001
	Reticulin 1	13 (21.7)	7 (23.3)	4 (5.8)	
	Reticulin 2	22 (36.7)	7 (23.3)	17 (24.6)	
	Reticulin 3	9 (15)	2 (6.7)	24 (34.8)	
	Reticulin 4	0 (0)	0 (0)	24 (34.8)	

***Note:*** Chi-square test was applied. P < 0.05 was statistically significant.

**Table-IV:** Mean blood counts at presentation in PV, ET, and PMF based on age groups.

			PV	ET	PMF
Age Groups	1–15 Years	WBC	.	.	8.5
		Hb	.	.	9
		HCT	.	.	23
		Plts	.	.	797
	> 15–30 Years	WBC	7.2	8	7.2
		Hb	17.7	13.7	10.9
		HCT	55	40	29
		Plts	186	1008	748
	> 30–50 Years	WBC	14	10	8.5
		Hb	16.8	13.4	10.7
		HCT	52	41	33
		Plts	706	1051	611
	> 50 Years	WBC	16.2	8.9	23.3
		Hb	17.9	12.2	10.8
		HCT	56	37	34
		Plts	562	863	611

***Note:*** WBC (10^9^/L), Hb (g/dL),HCT (%), Plts (billion cells/L).

**Table-V:** Mean blood counts at presentation in PV, ET, PMF based on gender.

			PV	ET	PMF
Gender	Females	WBC	14.5	8.8	13.7
		Hb	16.8	12.4	9.6
		HCT	52	37	29
		Plts	664	975	483
	Males	WBC	15.3	9	19.1
		Hb	17.8	13.2	11.3
		HCT	55	40	35
		Plts	580	930	718

***Note:*** WBC (109/L), Hb (g/dL), HCT (%), Plts (billion cells/L).

## DISCUSSION

This study represents the real-world data from 159 Pakistani Ph-negative MPN patients. The median age of presentation of these patients was 54 years, which is nearly a decade younger than reported worldwide^10^-^13^ but similar to findings from Malaysia.^14^ The mean age of patients with PV, ET, and PMF was 53, 47, and 51 years, respectively, which is younger than reported globally.^15^,^16^ There is a male predominance in our patients with MPN, which contrasts the internationally published data where PV is associated with male predominance while ET is seen primarily in females.^17^ This can be related to socioeconomic factors that cause more male patients to seek healthcare and tertiary hospital care than females, as seen in other diseases around the world.^18^

Table-IV outlines the differences in the baseline blood counts in MPNs based on age groups, and mean WBC count is higher in older age groups in both PV and PMF, while there is no difference in blood counts at presentation based on gender in these diseases. This study demonstrates a significant proportion of patients diagnosed with PV and ET have bone marrow fibrosis reticulin one or higher. Some studies suggest that bone marrow fibrosis at diagnosis is associated with an increased risk of fibrotic transformation, while others do not.^19^-^22^ At baseline, a small subset of patients with ET exhibited reticulin fibrosis. Although the WHO classification only allows a minor degree of reticulin fibrosis for diagnosing ET, the diagnosis was based on typical megakaryocytic morphology.^8^,^23^ It is unclear whether these cases represent ET with fibrosis caused by increased platelet-derived growth factors, co-existing pathologies, or pre-fibrotic MF or MPN-U at the outset. Allele burden measurement was not available at our institute, which could have helped diagnostic correlation of these patients, along with the degree of STAT phosphorylation of megakaryocyte nuclei and some other parameters; it is uncertain whether this would have led to any change in prognosis or treatment.^8^

None of our PV patients were found positive for JAK2 exon 12 mutation. A Study conducted by Latif et al.^24^ on MPN patients reported that none of the patients were found positive for JAK2 exon 12 mutation. However, study on Pakistani cohort of 24 PV patient by Akram et al.^25^, 19 were positive for JAK2 V617F and five were negative. Out of 5 JAK2 negative patients two were found positive for JAK2 exon 12 mutation on sequence analysis. Our results are quite comparable to results reported in previous studies on Pakistani population. Our cohort was found negative for c-MPL mutation. Studies conducted by Zaidi et al^26^ and Latif et al^24^ on ET and PMF patients reported that none of the patients were found positive for c-MPL mutation.

There is a need for further extended molecular profiling in triple negative patients of ET and PMF with next generation sequencing for ASXL1, TET2, DNMT3A, SRSF2, U2AF1, SF3B1, IDH1/IDH2, CBL, NRAS, KRAS and SETB1. The extended molecular profiling helps in triple negative patients for risk stratification, choosing therapeutic options and prognosis.

The presence of driver mutations (JAK2 V617F, JAK2 exon 12, CALR, or MPL) carries a strong impact on clinical course, disease phenotype, and prognosis. There is a higher incidence of thrombosis with JAK2V617F and is more aggressive disease outcome in PMF. Patients positive for CALR carries better prognosis and lower incidence of thrombosis. Double positive mutations e.g JAK2 + CALR may behave worse than single mutation. Patients with triple negative mutation carries worst outcome.

The anemia is a frequent complication of PMF, and the mean Hb in the PMF cohort was 10.7 g/dl. These cases can potentially represent post-PV myelofibrosis rather than true PMF, and they were probably classified as PMF because WHO criteria mandate a prior diagnosis of PV to qualify as this entity.^24^ In Pakistan, the lack of a structured health referral system may be responsible for delays in diagnosis and referrals, leading to the first presentation to a hematology facility with post-PV-myelofibrosis. Approximately one-fourth of PV or ET patients in this cohort presented with mild mucosal bleeding, which can be related to antiplatelet therapy or acquired von Willebrand disease. Still, none had severe or clinically significant bleeding.

Abdominal and microvascular symptoms were more common in PMF patients, particularly those over the age of 50 than in younger patients. Interestingly, arterial occlusive events included IHD in 8.2% of patients and CVA in 7.5%, too, in patients older than 50. This contrasts with other published data, which shows that the percentage is around 16.2%. This can be simply due to a lack of reporting of acute coronary disease, the protective effect of an antiplatelet or younger patient cohort.^27^

The results are similar to data from Pakistan.^6^,^7^ The MPL mutations were not present in any of our patients. Prior research has also shown that Pakistani patients have a very low incidence of MPL mutations. In a 2022 study, out of 50 patients, only three were found to be MPL positive.^28^ Similarly, in a research from Karachi, Pakistan, out of 137 patients, none were found to be MPL positive.^5^ JAK2 exon 12 was not detected in any of the our patients., which is comparable to previous studies. In a study from Lahore, only two JAK2V617F negative patients were found to have mutations in JAK2 exon 12.^25^ MPL and JAK2 exon 12 mutations were also not identified in a study from Rawalpindi.^29^ However, the frequency of JAK2 mutation in PV, ET, and PMF patients is lower than reported worldwide. A number of genetic, diagnostic, epidemiological, and healthcare-related factors may contribute to Pakistan’s reported lower incidence rates of JAK2 exon 12, MPL, and CALR mutations as compared to the West. Certain mutations may naturally occur at lower frequencies in the Pakistani population as a result of environmental or evolutionary reasons. The true mutation frequencies at country level may not be captured by the limited sample sizes of many Pakistani studies cohort, which frequently come from a single hospital or area. Patients usually get diagnosed late in the course of the illness, and MPNs are frequently misdiagnosed as other diseases (such as idiopathic thrombocytosis, anemia, or infections). Inadequate molecular profiling can lead to cases being overlooked or mislabeled as “triple negative.” Western countries regularly screen for mutations and adhere to stringent diagnostic standards (WHO/ELN). Variability in laboratory quality and reporting standards, as well as inconsistent use of guidelines, may exist in Pakistan.

The mutational profile data of Pakistani MPN patients has been expanded by this work. These genetic tests were established at our center. Additionally, it emphasized how Pakistani MPN patients’ mutational landscape differs greatly from that of patients in other countries. This highlights the necessity of doing larger, multi-center cohort studies.

### Strengths of study:

- Improved understanding of pathogenesis and mutational profile of Philadelphia negative MPNs in Pakistani population.
- Establishment of lab guidelines: This study enabled us to establish guidelines for detection of JAK2 V617F, JAK2 Exon 12, CALR and C-mpl in the molecular laboratory with limited resources. Accurate detection methods for molecular mutations were established in molecular laboratory where associated clinics are continuously monitoring the impact of molecular mutations over disease outcome.
- Association with clinical characteristics: This study highlighted the association of clinical characteristics of Ph negative MPNs with mutational profile and outcome.

### Limitations:

It includes a single-center study. Second, it did not address specific issues in MPNs, particularly fertility and pregnancy outcomes, associated cardiovascular risk factor assessments, and a lack of resources for improved risk stratification and response assessment per the International Working Group on MPN guidelines.

## CONCLUSIONS

This study highlights the molecular landscape of Philadelphia-negative MPNs in the Pakistani population, demonstrating a comparatively lower frequency of JAK2 V617F, JAK2 exon 12, CALR, and MPL mutations than typically reported in Western cohorts. While JAK2 V617F remains the most common mutation. Similarly, there was a lack of representation for CALR and MPL mutations, which are especially important in PMF and ET. In addition to suggesting a potential impact of diagnostic restrictions, such as limited access to sophisticated molecular testing, underdiagnosis, and small, non-representative sample sizes, these findings may also reflect underlying genetic variation. Further research employing next-generation sequencing and larger molecular panels is necessary due to the significant percentage of triple-negative cases.

### Author’s contribution:

**MAK:** Did data collection, statistical analysis, data interpretation, manuscript writing and is responsible for the integrity of the research. **SA:** Conceived and supervised the study project and approved the manuscript writing. **MAS, MK, MK and ZA:** Did revision and editing of the manuscript. All authors have read and approved the final version of the manuscript.
